# Evaluation of Ultra-High Molecular Weight Polyethylene (UHMWPE) Implants in Orbital Fracture Reconstruction: A Multicenter Cohort Study

**DOI:** 10.1007/s12663-025-02618-w

**Published:** 2025-07-05

**Authors:** Konstantinos Afxentiou, Raimund H. M. Preidl, Marco R. Kesting, Adem Aksu, Achim von Bomhard, Katharina Pippich, Stefanie Wilhelm, Rainer Lutz

**Affiliations:** 1 Department of Oral and Maxillofacial Surgery, University of Erlangen-Nuremberg, Erlangen, Germany; 2 Friedrich-Alexander-Universität Erlangen-Nürnberg (FAU), Erlangen, Germany; 3 KLS Martin SE & Co. KG, Tuttlingen, Germany; 4 Department of Maxillofacial Surgery, Technical University of Munich, Munich, Germany; 5 Technical University of Munich, Munich, Germany

**Keywords:** Ultra/high molecular weight polyethylene, Orbital floor fractures, Orbital volumes, marPOR

## Abstract

**Purpose:**

Orbital wall reconstruction remains a challenge in reconstructive CMF surgery due to the delicate anatomical structures and their potential functional impairment due to inefficient treatment. The use of biomaterials in reconstructive surgery has revolutionised the therapy in this area. Ultra-high molecular weight polyethylene (UHMWPE) has shown promising results due to its biocompatibility, mechanical properties, and versatility. This paper presents the first clinical study of the use of UHMWPE in orbital fracture reconstruction.

**Methods:**

In a multicentre prospective study consisting of 50 patients undergoing orbital floor reconstruction, UHMWPE implants were used to reconstruct the orbital volume after fracture. 46 Patients underwent preoperative and postoperative 3D radiography and orbital volume segmentation.

**Results:**

A significant postoperative volume reduction of the affected orbits was observed in the entire cohort (*p* < 0.001) with a significant alignment to the healthy contralateral orbits. There were no implant-related complications.

**Conclusion:**

Orbital wall reconstruction with UHMWPE (marPOR) implants is a reliable method to restore adequate orbital volume for functional and aesthetic recovery in trauma patients.

## Introduction

Facial trauma is often associated with orbital fractures due to the delicate anatomical structures of the orbit [1]. Consequently, orbital fractures require prompt and precise surgical intervention to restore orbital volume and support the orbital soft tissues to prevent complications such as diplopia, enophthalmos, or even vision loss [2]. Traditional methods of orbital fracture repair include the use of autogenous bone grafts [3, 4], titanium implants [5] and absorbable plates [6]. However, these techniques have limitations such as donor site morbidity, implant palpability, and difficulty in contouring to the patient’s anatomy.

In recent years, there has been a growing interest in the use of biomaterials in surgery, driven by advances in materials science, surgical techniques, and patient outcomes research. Among these biomaterials, ultra-high molecular weight polyethylene (UHMWPE) has emerged as a promising alternative due to its unique combination of mechanical properties and biocompatibility. Its highly porous structure and long polymer chains contribute to its wear resistance and low coefficient of friction, making it suitable for applications involving repetitive sliding movements, in this case the ocular muscles, and providing optimal conditions for potential vascularization and osteointegration. With a molecular weight ranging from 3.5 to 7.5 million g/mol, UHMWPE exhibits exceptional strength and toughness [7]. In the context of orbital fracture reconstruction, its customizability allows precise replication of the patient’s anatomy, ensuring optimal fit and contour of the implant. In addition, its mechanical strength provides sufficient support for the orbital contents while allowing for physiological movement, minimizing the risk of implant failure or dislocation seen with other biomaterials, including the widely used polydioxanone sheets [8]. These properties make it an attractive choice for craniofacial implants. The present study is the first multicentre study to provide a comprehensive overview of orbital fracture reconstruction in trauma patients using UHMWPE implants.

## Materials and Methods

### Study Design and Selection Process

The study protocol was reviewed and approved by the Ethics Committees of the Friedrich-Alexander University of Erlangen (approval no.: 21-413-B), the Technical University of Munich (approval no.: 737/21 S) and the State of Baden-Württemberg (approval no.: B-F-2022-031). Written informed consent was obtained from all participants prior to enrolment.

Adult patients with orbital fractures presenting to the centres between August 2022 and August 2023 were screened and eligible patients were informed about the study. Patients were excluded if: (1) any non-medically managed serious systemic disease, (2) immunosuppression status, (3) previous radiotherapy in the respective surgical field, (4) recent history of substance abuse (i.e. excessive use of or dependence on recreational drugs, alcohol) that would preclude reliable assessment, (5) pregnancy or women planning to become pregnant within the study period, (6) prisoners (because follow-up cannot be guaranteed), (7) participation in any other medical device or drug study within the previous month that could influence the results of this study, (8) skeletally immature.

All eligible patients underwent a comprehensive pre-operative evaluation, including a detailed medical history, physical examination and imaging studies. Predicted drop-outs were taken into account to determine the final sample size required for the study.

### Surgical Procedure and Postoperative Care

**Fig. 1:**
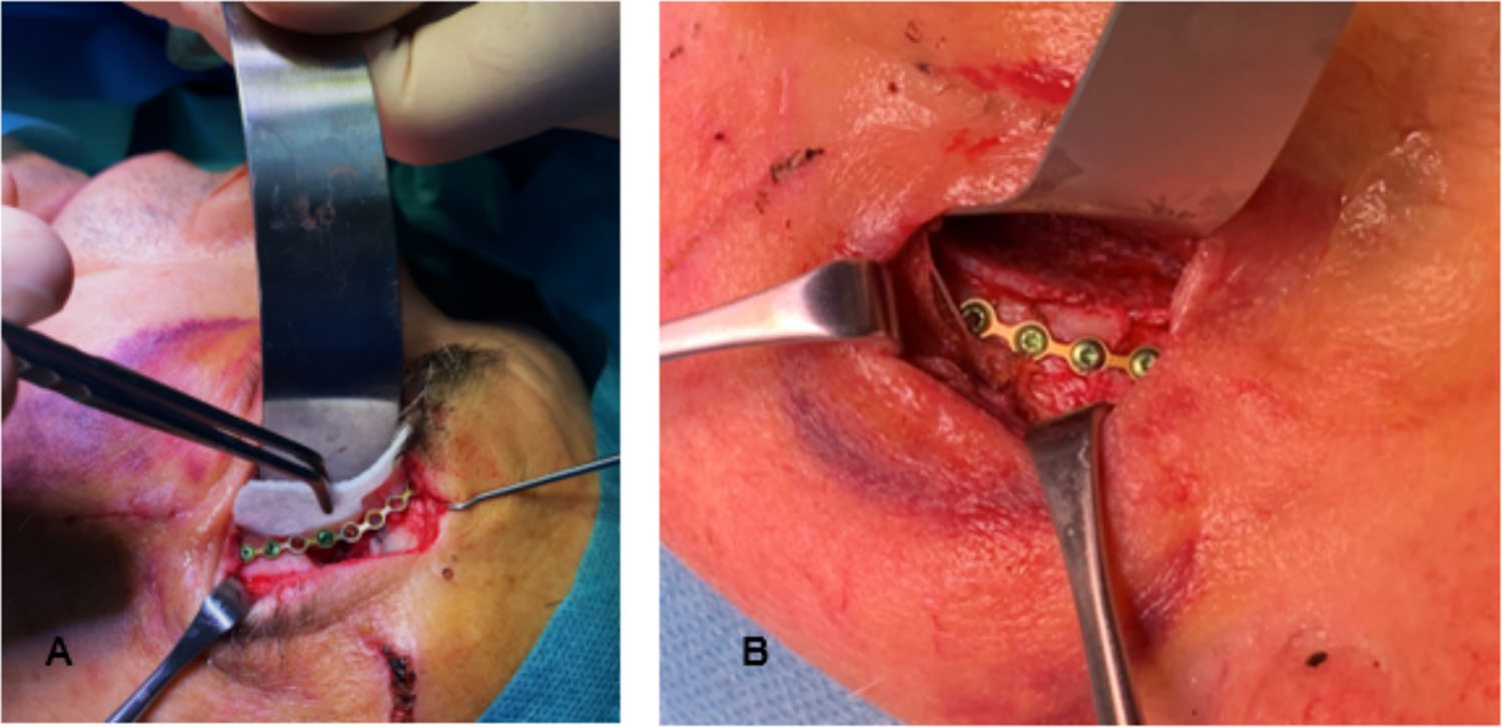
Intra-operative photographs showing marPOR implant placement through a lateroorbital incision (**A**), and final implant position (**B**)

Surgical procedures were performed by experienced and specialized craniofacial surgeons under general anaesthesia according to standardized protocols and techniques (Fig. 1). The choice of surgical approach (transcutaneous in the periorbital region or transconjunctival) was determined based on the location and severity of the orbital fracture, patient age and preoperative swelling.

After exposure of the fracture site, the UHMWPE implants were modified to achieve anatomical reduction, restore orbital volume, and preserve soft tissue integrity. Haemostasis was achieved, and surgical incisions were closed using techniques appropriate to the surgical approach. Patients received standardized post-operative care, including antibiotics and analgesia.

Patients were closely monitored throughout their hospital stay for signs of complications, including infection, bleeding, or wound dehiscence. Ocular function and neurological status, such as hypesthesia of the V2 nerve, were also assessed. Follow-up visits were scheduled to assess the postoperative recovery and to monitor for any implant-related complications.

### Image Acquisition and Volumetric Analysis

A total of 46 patients underwent preoperative 3D imaging (CT or CBCT scans) to assess fracture severity and location. Post-operative imaging was performed as clinically indicated to assess implant position and fracture reduction in the same manner. The scans were then reconstructed as axial, coronal, and sagittal slices and transferred to a three-dimensional imaging software program (3D Slicer ver. 5.6.1, https://www.slicer.org/) [9].

**Fig. 2:**
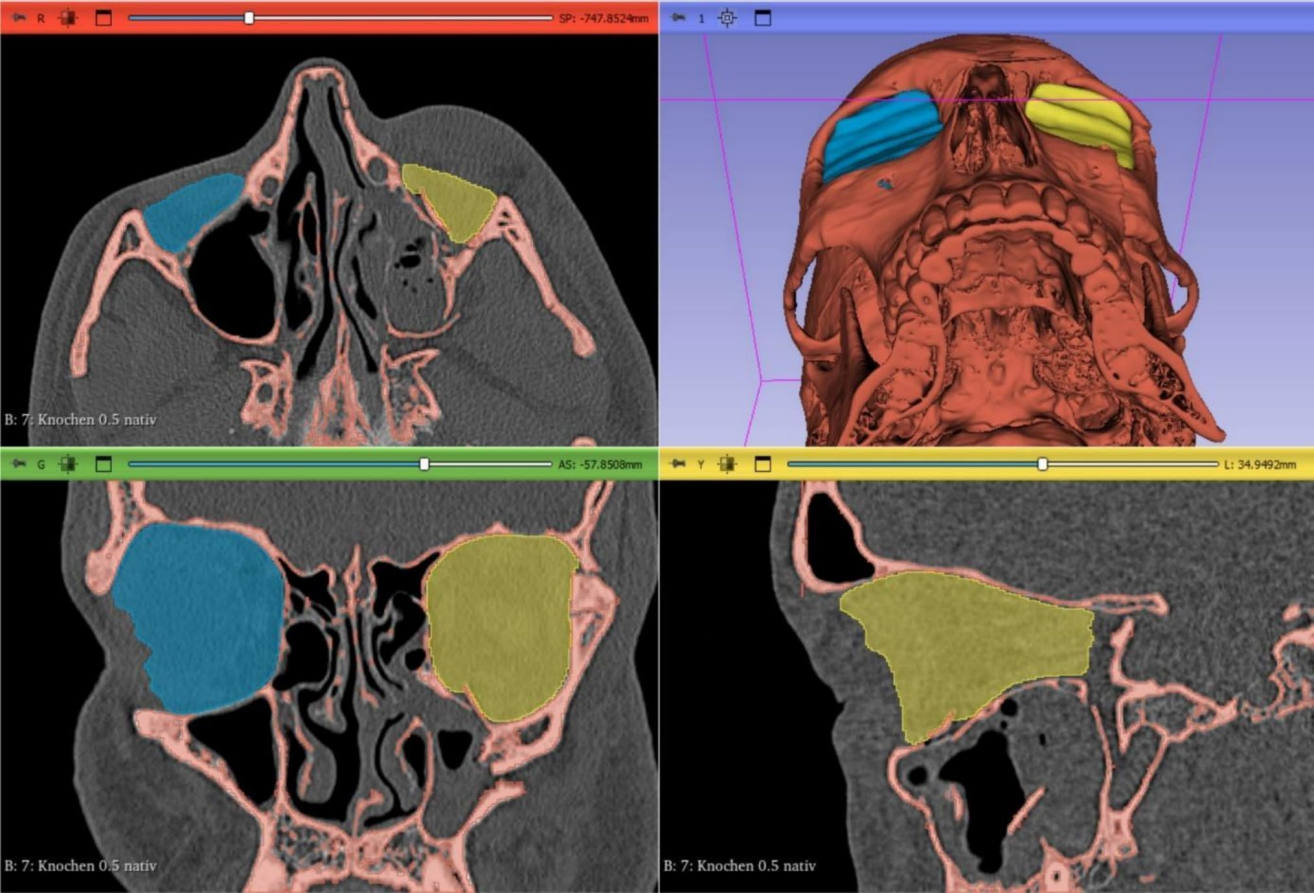
Preoperative CT scan of the midface in axial (**A**), coronal (**C**), sagittal (**D**) and 3D reconstruction (**B**). Segmented volumes of fractured (yellow) and intact orbit (blue) were generated using 3D-Slicer software

**Fig. 3:**
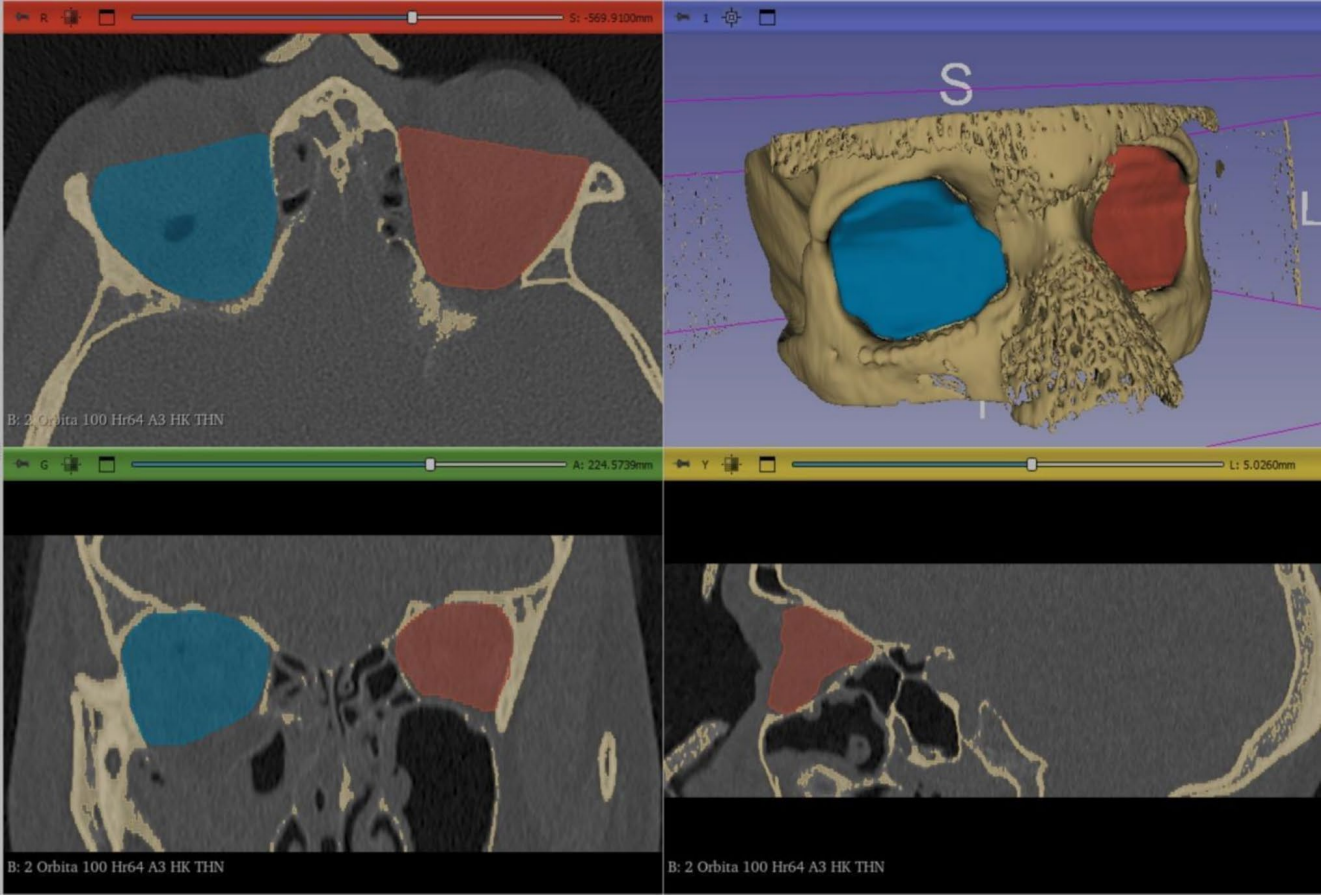
Postoperative CT scan of the midface in axial (**A**), coronal (**C**), sagittal (**D**) and 3D reconstruction (**B**). Segmented volumes of the fractured and reconstructed (blue) and intact orbit (red) were generated using 3D-Slicer software

The following anatomical landmarks were used to segment the orbital volume as previously described [10]: the anterior orbital rim was defined as a straight line connecting the posterior surface of the lacrimal sulcus and the lateral orbital rim. The posterior margin was defined as the orbital apex excluding the optic canal. The herniated orbital contents were included in the measurement of the orbital volume. Volumetric analysis was performed by an independent third-party analyst (m3i GmbH – I.Z.) who was not involved in the study design or had contact with the study patients. Software calibration was utilized to further adjust the contrast in the CT images to ensure accurate marking of the anatomical landmarks, and a manual segmentation as per standardized measurement protocols was carried out [11]. Additionally, a specialist in each center, blindly verified the volumetric measurements to further increase the reliability and eliminate any potential bias. The resulting volume was calculated for each orbit in cm^3^ and a three-dimensional image was generated (Figs. 2 and 3).

**Fig. 4:**
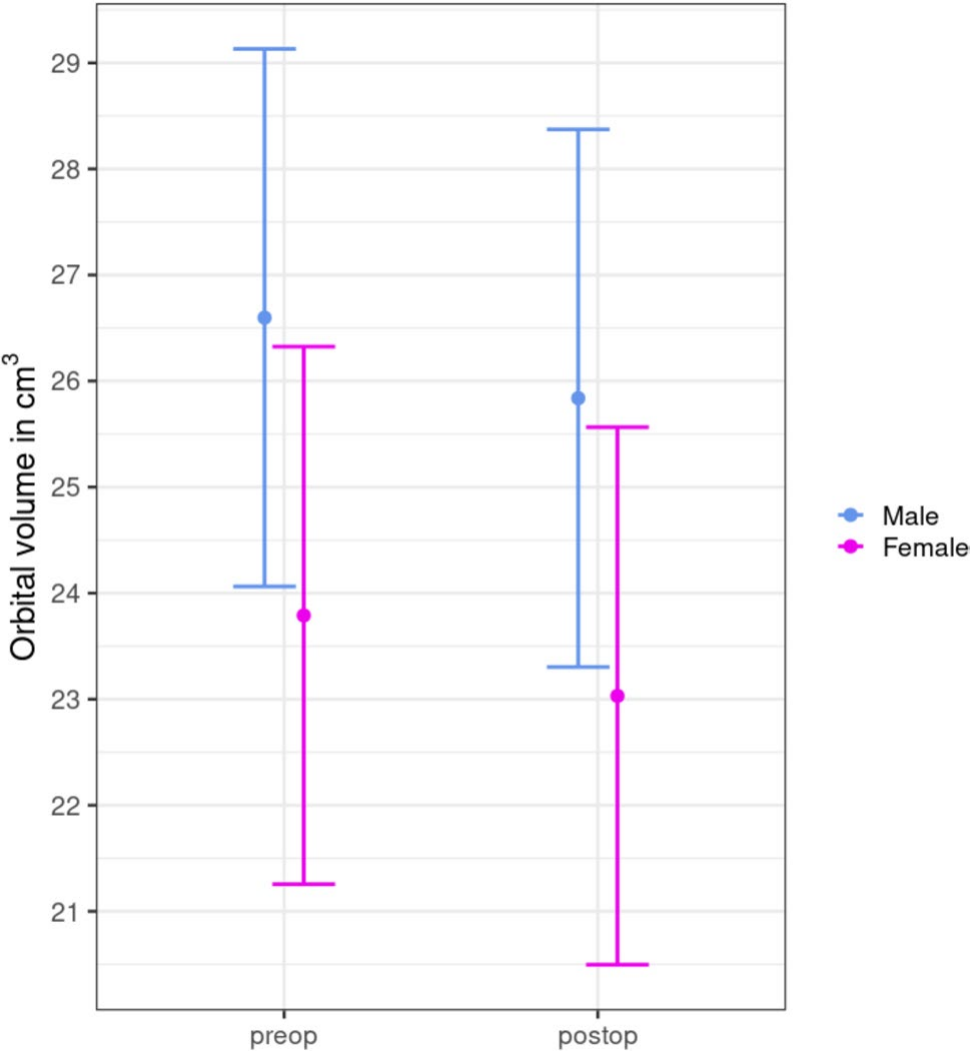
Predicted orbital volume pre- and post-operatively for male and female patients. There is a significant mean volume reduction in both groups

Data were analysed using paired difference t-test and linear mixed model to generate the following graphs, as shown in Fig. 4.

## Results

A total number of 50 patients were included in this study, 22 females and 28 males. The age ranged from 21 to 97 years, with a mean age of 56.8 years. Fractures involved the right orbit in 29 patients, the left orbit in 19 patients and both orbits in 2 patients. 46 (92%) of the fractures were orbital floor fractures, 1 fracture was an isolated medial wall fracture and the remaining 3 fractures were isolated orbital roof fractures. Zygomatic involvement was documented in 34 cases (68%). On the first postoperative day, 7 patients had unremarkable local findings, 2 patients reported local irritation of the affected area, and 40 patients had swelling. Only one patient had significant swelling due to dual antiplatelet therapy. No major complications such as bleeding, surgical site infection, implant dislocation or visual disturbances were documented during the immediate post-operative hospital stay. 4 patients failed to undergo a post-operativ imaging thus were not incluced in the volumetric analysis.

The mean volume ± standard deviation (SD) of all injured orbits before surgery was 23.64 ± 2.69 cm^3^ for female patients and 26.47 ± 3.50 cm^3^ for male patients. The mean orbital volume (OV) ± standard deviation (SD) of the reconstructed orbits was 22.92 ± 2.67 cm^3^ for female patients and 25.66 ± 3.35 cm^3^ for male patients. The mean OV ± (SD) of the intact orbits before surgery was 23.04 ± 2.56 cm^3^ for female patients and 25.39 ± 3.32 cm^3^ for male patients. Postoperatively, the mean OV ± (SD) was 22.77 ± 2.51 and 25.26 ± 3.13 cm^3^, respectively. The mean absolute volume difference ± (SD) of the fracture site compared to the reconstructed orbit was 1.15 ± 0.99 cm^3^ in female patients and 1.16 ± 0.72 cm^3^ in male patients. In comparison, the mean absolute difference of the healthy OV was 0.61 ± 0.53 cm^3^ in females and 0.63 ± 0.53 cm^3^ in males.

Considering these results, the difference between the orbital volume of the affected site preoperatively and the reconstructed orbit was highly statistically significant (p < 0.001), whereas the difference on the healthy site preoperatively and postoperatively was not statistically significant (p = 0.54).

## Discussion

This study presents the first multicentre cohort of patients with adequately reconstructed orbital fractures after trauma using UHMWPE implants. We aimed to evaluate the outcome of orbital reconstruction with Ultra High Molecular Weight Polyethylene (UHMWPE) implants by measuring the difference in orbital volume before and after the surgery in a clinical setting.

Orbital fractures represent a significant subset of craniofacial injuries [12, 13], and while small fractures can be managed conservatively, surgical intervention is often required to restore orbital anatomy and function [14]. Although the ideal material for reconstruction remains controversial, the following characteristics are widely accepted to provide the best results. The material should be biocompatible and non-carcinogenic. It should conform to the patient’s anatomy and retain its new shape. If alloplastic, the material should be inexpensive and readily available. It should not promote microbial growth or be allergenic, and it should be rigid enough to support the orbital contents.

Autogenous implants remain a viable option due to their biocompatibility, but donor site morbidity, harvesting time, and cosmetic concerns are some of the drawbacks that limit their potential [15, 16].

There are a variety of alloplastic materials available for orbital floor reconstruction, but to date, none seems to combine all of the above characteristics to make it an ideal material [17, 18]. PDS and Polyglactin 910/PDS (Ethisorb) patches, two widely used materials, have been shown to be associated with increased infection rates, as well as inferior globe position and progressive enophthalmos due to their flexible nature, thus recommending overcorrection of the globe during surgery [8, 19, 20]. Therefore, PDS is preferred for reconstruction of small defects [21] and 910/PDS for small to moderate defects (maximum 2 × 2 cm) [22]. Polypropylene mesh, a material commonly used in abdominal surgery that can be both resorbable and non-resorbable, is a viable alternative for medium-sized orbital floor fractures, but studies report persistent diplopia and enophthalmos in some cases [23, 24]. Titanium mesh, a stiffer and more stable material, has traditionally been used for larger defects [25, 26], but orbital adhesion with subsequent ocular motor dysfunction has been reported [27–29], and it interferes with radiographic examination and causes weathering sensations due to its metallic nature [30].

UHMWPE is a polymer that differs from the more familiar high-density polyethylene (HDPE) in terms of molecular weight and average chain length. In addition to its mass, the molecular structure of the polymer plays an important role in its physical and chemical properties. Its interconnected pore network allows rapid infiltration by the host tissue and promotes osteointegration, resulting in a reduced risk of implant migration and excellent long-term stability [7]. A meta-analysis of case series studies by Schellini et al. highlighted the advantages of porous polyethylene implants in the orbital region, demostrating a lower risk of implant exposure [31]. The extensive use of UHMWPE in weight-bearing anatomical regions such as hip and knee arthroplasty undoubtedly demonstrates its toughness and biocompatibility [32]. Therefore, it’s mechanical strength provides sufficient support for the orbital contents, yet its thermoplastic nature ensures optimal conformability to the patient’s anatomy [33, 34], resulting in precise orbital reconstruction. Our clinical results mirror those of our previous study in cadavers, with favourable results in terms of orbital volume restoration and functional improvement [10].

There are several potential complications that may arise due to the non-resorbable character of the implant, such as implant migration or long-term tissue response. From the three most popular alloplastic materials (porous polyethylene sheets PPE, Polypropylene mesh and Titanium implants), studies report superior outcomes with fewer complications using porous polyethylene sheets like marPOR-implant. Ghafar et al. studied 24 patients who received PPE sheets for reconstruction of orbital floor fractures, none of whom required reoperation or showed Signs of implant displacement [35]. Lupi et al. used UHMWPE in 32 patients with no implant migration, extrusion or enophthalmos [36]. Krishna et al. used PPE implant in 12 patients with fractures of the orbital floor and within the 1-year follow-up no implants were extruded and there were no signs of inflammatory reactions [37]. While our study demonstrated the effectiveness of UHMWPE implants to restore the volume in orbital fracture reconstruction and although no major complications were observed during the immediate post-operative period, a long-term follow-up is required to further ensure the implants overall efficacy.

The main limitation of this study is the use of only one specific orbital implant; therefore, no comparison between different implants can be made. Additionally, its radiolucency can be proven challenging to determine the exact position of the implant. While the immediate post-operative CT scans in this study allowed a sufficient differentiation due to the contrast with the surrounding bone and post-operative emphysema and oedema around the implant, future research could explore modifications to the implant material, such as the incorporation of radio-opaque additives to enhance its visibility and allow long-term monitoring.

Despite these considerations, the results of this study support the short-term safety of UHMWPE implants and concludes that marPOR-Implant is a reliable alternative material for orbital fracture reconstruction. UHMWPE offers several distinct advantages such as long-term stability, exceptional strength, and biocompatibility. In addition, it is easy to handle, contour, and process to the patient’s unique anatomy, resulting in accurate orbital volume restoration.

## References

[1] Boffano P et al (2015) European Maxillofacial Trauma (EURMAT) project: a multicentre and prospective study. J Craniomaxillofac Surg 43(1):62–70 25457465 10.1016/j.jcms.2014.10.011

[2] Schönegg D et al (2018) Correlation between increased orbital volume and enophthalmos and diplopia in patients with fractures of the orbital floor or the medial orbital wall. J Craniomaxillofac Surg 46(9):1544–1549 30041991 10.1016/j.jcms.2018.06.008

[3] Kosaka M et al (2004) Orbital wall reconstruction with bone grafts from the outer cortex of the mandible. J Craniomaxillofac Surg 32(6):374–380 15555521 10.1016/j.jcms.2004.06.006

[4] Garg V, Giraddi GB, Roy S (2015) Comparison of efficacy of mandible and iliac bone as autogenous bone graft for orbital floor reconstruction. J Maxillofac Oral Surg 14(2):291–298 26028849 10.1007/s12663-014-0654-4 PMC4444722

[5] Chattopadhyay C et al (2022) Reconstruction of orbital floor fractures with titanium micromesh: our experience. J Maxillofac Oral Surg 21(2):369–378 35712422 10.1007/s12663-020-01407-x PMC9192857

[6] Gierloff M et al (2012) Orbital floor reconstruction with resorbable polydioxanone implants. J Craniofac Surg 23(1):161–164 22337397 10.1097/SCS.0b013e3182413edc

[7] Hussain M et al (2020) Ultra-High-Molecular-Weight-Polyethylene (UHMWPE) as a promising polymer material for biomedical applications: a concise review. Polymers (Basel) 12(2):323 32033140 10.3390/polym12020323 PMC7077409

[8] Park HY et al (2022) Quantitative assessment of increase in orbital volume after orbital floor fracture reconstruction using a bioabsorbable implant. Graefes Arch Clin Exp Ophthalmol 260(9):3027–3036 35262763 10.1007/s00417-022-05610-z

[9] Fedorov A et al (2012) 3D Slicer as an image computing platform for the quantitative imaging network. Magn Reson Imaging 30(9):1323–1341 22770690 10.1016/j.mri.2012.05.001 PMC3466397

[10] Foerster Y et al (2022) Ultra-high molecular weight polyethylene (marPOR) is a suitable material for the reconstruction of orbital floor fracture defects in human cadavers. J Maxillofac Oral Surg 23:1646 39618457 10.1007/s12663-022-01789-0 PMC11607238

[11] Chepurnyi Y et al (2020) Reliability of orbital volume measurements based on computed tomography segmentation: validation of different algorithms in orbital trauma patients. J Cranio-Maxillofac Surg 48(6):574–581 10.1016/j.jcms.2020.03.007 32291132

[12] Nakamura T, Gross CW (1973) Facial fractures. Analysis of five years of experience. Arch Otolaryngol 97(3):288–290 4696044 10.1001/archotol.1973.00780010296016

[13] Gwyn PP et al (1971) Facial fractures–associated injuries and complications. Plast Reconstr Surg 47(3):225–230 5101680 10.1097/00006534-197103000-00004

[14] Kim HS, Jeong EC (2016) Orbital floor fracture. Arch Craniofac Surg 17(3):111–118 28913267 10.7181/acfs.2016.17.3.111 PMC5556798

[15] Kontio R (2004) Treatment of orbital fractures: the case for reconstruction with autogenous bone. J Oral Maxillofac Surg 62(7):863–868 15218567 10.1016/j.joms.2004.03.003

[16] Bande CR et al (2015) Reconstruction of orbital floor fractures with autogenous bone graft application from anterior wall of maxillary sinus: a retrospective study. J Maxillofac Oral Surg 14(3):605–610 26225051 10.1007/s12663-014-0716-7 PMC4511894

[17] Shetty P et al (2009) Options in orbital floor reconstruction in blowout fractures: a review of ten cases. J Maxillofac Oral Surg 8(2):137–140 23139492 10.1007/s12663-009-0034-7 PMC3453935

[18] Bourry M et al (2021) Clinical evaluation of the efficacy of materials used for primary reconstruction of orbital floor defects: Meta-analysis. Head Neck 43(2):679–690 33145908 10.1002/hed.26518

[19] Jank S et al (2003) Orbital floor reconstruction with flexible Ethisorb patches: a retrospective long-term follow-up study. Oral Surg Oral Med Oral Pathol Oral Radiol Endod 95(1):16–22 12539022 10.1067/moe.2003.11

[20] Iizuka T et al (1991) Reconstruction of orbital floor with polydioxanone plate. Int J Oral Maxillofac Surg 20(2):83–87 1904906 10.1016/s0901-5027(05)80712-x

[21] Kos M, Brusco D, Engelke W (2006) Results of treatment of orbital fractures with polydioxanone sheet. Polim Med 36(4):31–36 17402230

[22] Büchel P et al (2005) Reconstruction of orbital floor fracture with polyglactin 910/polydioxanon patch (ethisorb): a retrospective study. J Oral Maxillofac Surg 63(5):646–650 15883939 10.1016/j.joms.2004.11.013

[23] Tuncer S et al (2007) Reconstruction of traumatic orbital floor fractures with resorbable mesh plate. J Craniofac Surg 18(3):598–605 17538325 10.1097/01.scs.0000246735.92095.ef

[24] Touil H et al (2020) Reconstruction of orbital floor fractures with Polypropylen mesh. Tunis Med 98(1):49–54 32395777

[25] Degala S, Shetty SK, Biddappa L (2013) Reconstruction of post-traumatic internal orbital wall defects with titanium mesh. J Maxillofac Oral Surg 12(4):418–423 24431881 10.1007/s12663-012-0444-9 PMC3847028

[26] Banica B et al (2013) Titanium preformed implants in orbital floor reconstruction - case presentation, review of literature. Maedica (Bucur) 8(1):34–39 24023596 PMC3749759

[27] Shah HA et al (2018) Extra-ocular movement restriction and diplopia following orbital fracture repair. Am J Otolaryngol 39(1):34–36 28969869 10.1016/j.amjoto.2017.08.008

[28] Lee HB, Nunery WR (2009) Orbital adherence syndrome secondary to titanium implant material. Ophthalmic Plast Reconstr Surg 25(1):33–36 19273920 10.1097/IOP.0b013e3181929b6e

[29] Kersey TL et al (2013) Orbital adherence with titanium mesh floor implants: a review of 10 cases. Orbit 32(1):8–11 23387447 10.3109/01676830.2012.736597

[30] Holtmann H et al (2016) Orbital floor fractures–short- and intermediate-term complications depending on treatment procedures. Head Face Med 12:1 26729217 10.1186/s13005-015-0096-3 PMC4700729

[31] Schellini S et al (2016) Porous and nonporous orbital implants for treating the anophthalmic socket: a meta-analysis of case series studies. Orbit 35(2):78–86 26928263 10.3109/01676830.2016.1139591

[32] Bistolfi A et al (2021) Ultra-high molecular weight polyethylene (UHMWPE) for hip and knee arthroplasty: the present and the future. J Orthop 25:98–106 33994706 10.1016/j.jor.2021.04.004 PMC8102204

[33] Kozakiewicz M et al (2013) Technical concept of patient-specific, ultrahigh molecular weight polyethylene orbital wall implant. J Craniomaxillofac Surg 41(4):282–290 23333489 10.1016/j.jcms.2012.10.007

[34] Ahmad M et al (2012) Mechanical, rheological, and bioactivity properties of ultra high-molecular-weight polyethylene bioactive composites containing polyethylene glycol and hydroxyapatite. ScientificWorldJournal 2012:474851 22666129 10.1100/2012/474851 PMC3361308

[35] Abd El Ghafar AE et al (2025) Long-term clinical outcomes of isolated orbital floor fracture reconstruction using nonresorbable implants. Indian J Ophthalmol 73(2):191–198 39853138 10.4103/IJO.IJO_1100_24 PMC11991547

[36] Lupi E et al (2004) Orbital floor repair using MEDPOR porous polyethylene implants. Invest Ophthalmol Vis Sci 45(13):4700–4700

[37] Sai Krishna D, Soumadip D (2016) Reconstruction of orbital floor fractures with porous polyethylene implants: a prospective study. J Maxillofac Oral Surg 15(3):300–307 27752198 10.1007/s12663-015-0840-z PMC5048316

